# Prevalence of Depression and Anxiety Symptoms of High School Students in Shandong Province During the COVID-19 Epidemic

**DOI:** 10.3389/fpsyt.2020.570096

**Published:** 2020-12-21

**Authors:** Zeng Zhang, Ailing Zhai, Mingchuan Yang, Junqing Zhang, Haotian Zhou, Chuanming Yang, Shanshan Duan, Cong Zhou

**Affiliations:** ^1^Jining Psychiatric Hospital, Jining, China; ^2^Jining Yucai High School, Jining, China; ^3^The First High School of Jiaxiang, Jiaxiang, China; ^4^University of Mississippi, Oxford, MS, United States; ^5^School of Mental Health, Jining Medical University, Jining, China; ^6^Department of Psychiatry, The First Affiliated Hospital of Kunming Medical University, Kunming, China

**Keywords:** COVID-19, high school students, depression, anxiety, mental health

## Abstract

**Background:** The coronavirus disease 2019 (covid-19) has brought physical risks as well as psychological challenges to the whole world. High school students are a special group suffering from both the academic pressure and the threat of the epidemic. The present study aims to conduct an online survey to investigate the psychological status of high school students in Shandong Province.

**Methods:** Using a web-based cross-sectional survey, data was collected from 1,018 voluntary high school students assessed with demographic information, the Patient Health Questionnaire-9 (PHQ-9), the Generalized Anxiety Disorder-7 (GAD-7) and a self-designed online-study effect survey. Correlation analysis was performed to explore the relationships between depression symptoms, anxiety symptoms, and study effect.

**Result:** The prevalence of depressive symptoms, anxiety symptoms, and a combination of depressive and anxiety symptoms was 52.4, 31.4, and 26.8%, respectively, among high school students in Shandong Province during the COVID-19 epidemic. And from moderate to severe severity level, the rates of depressive symptoms and anxious symptoms were 17.6 and 4.6%. Female students exhibited a higher rate and severity of mental symptoms than male, and grade one senior high school students got a higher rate and severity of mental symptoms than the other two grades. Nearly half of the students were not satisfied with their online-study effect. The PHQ-9 score had a strong positive correlation with the GAD-7 score. Both the PHQ-9 score the GAD-7 score had a negative correlation with the study-effect survey score.

**Conclusion:** Quite a number of high school students suffered from depression and anxiety symptoms during the COVID-19 epidemic. Sufficient attentions should be paid, and necessary supports should be provided, to protect the mental health of this special group.

## Introduction

In January 2020, the coronavirus SARS-CoV-2 was identified as the cause of an outbreak of severe pneumonia, and was officially designated as the coronavirus disease 2019 (covid-19) by the World Health Organization (1). This public health emergency has been escalating and threatening the welfare of society and human beings globally. The spread of COVID-19 pandemic has swept across 210 countries and territories with over 3 million cases and 210 000 deaths reported by April 30th, 2020 (https://covid19.who.int/). Apart from the impact on physical condition, there is also evidence that the direct and indirect psychological and social effects of the COVID-19 pandemic are pervasive and could affect mental health now and in the future (2).

The SARS-CoV-2 may minimally infect children and adolescents (1), and even if get infected, they seem to experience less severe COVID-19 than adults, with few or no symptoms (3, 4). Generally, children and adolescents are healthy and do not require much health care outside of regular checkups and immunizations (5). However, a healthy mental state is very important for children and adolescents. Globally, depression is the fourth leading cause of disease and disability among adolescents aged 15–19 years, and the 15th for those aged 10–14 years (6). A meta-analysis of the prevalence of depressive symptoms in children and adolescents in China indicated that the reported point prevalence of depressive symptoms ranged between 4 and 41%, the pooled prevalence of depressive symptoms was 19.85% (7). In the meantime, anxiety is the ninth leading cause of disease and disability for adolescents aged 15–19 years and sixth for those aged 10–14 years globally (6). Previous Chinese studies have shown that the incidence of anxiety symptoms among Chinese adolescents ranges from 13.7 to 24.5% (8, 9).

High school students (usually aged 15–18 years old) in China are a special group. The Chinese National College Entrance Exam, known as “GaoKao,” is the most important and the only criterion for entrance to Chinese universities, generating many depressive and anxious feelings to high school students, especially those grade three students (who are about to undergo this important test). The COVID-19 pandemic may worsen existing mental health problems among children and adolescents because of the unique combination of the public health crisis, social isolation, and economic recession (5). Furthermore, China has implemented country-wide school closures for over 3 months to prevent the spread of the epidemic. Students at all stages were home quarantined and could only accept online-study. Most of the students were more used to studying at school during their whole student career. They were hardly familiar with online study before. The changes of study environment and uncertain online-study effect may affect the students' mentality.

With the epidemic gradually kept under control, as of the start time of this research (May 1st), grade three high school students had been back to school for 2 weeks, while the other two grades were still in quarantine. Though the GaoKao has been postponed from June to July due to the COVID-19, the mental health of grade three students deserves to be concerned. The psychological status of grade one and grade two students should not be ignored, either. To our best knowledge, few studies have focused on the psychological health of high school students in China during the COVID-19 epidemic. An online mental health survey on ordinary Chinese people indicated that adolescents had a higher incidence of depressive symptoms during COVID-19 than adults. Zhou et al. (10) conducted an online survey among Chinese students aged 12–18 years, and found that the prevalence of depressive symptoms, anxiety symptoms, and a combination of depressive and anxiety symptoms was 43.7, 37.4, and 31.3%, respectively, and female gender and higher grade might be risk factors for depressive and anxiety symptoms. In this present study, we aimed to concentrate our attention on the mental health as well as online-study effect of senior high school students in Shandong Province. We speculated that students with different genders and different grades would exhibit distinct psychological status.

## Materials and Methods

### Subjects

We used a convenience sampling method to collect data in three high schools in Shandong Province from May 1st to May 7th, 2020. An online survey was conducted using a self-administered questionnaire delivered through the internet. The inclusion criterion was: high school students who voluntarily participate in the mental health assessments. Exclusion criteria were as follows: (1) present or previous history of other psychiatric or neurological illness or serious physical disease, (2) not in Shandong Province.

### Measurement Tools

By using the questionnaire, we have obtained demographic and neuropsychological data from the respondents.

1. General demographic information: Basic information including grade, age, gender, current residence, and history of close contact to SARS-CoV-2 were acquired. This study was set to anonymous to protect the privacy of the students.
2. The Patient Health Questionnaire 9-item (PHQ-9): The PHQ-9 is used to measure depressive symptoms. PHQ-9 is a simple and efficient self-assessment tool for depression screening based on DSM-IV (11). Participants are asked to report the presence of nine problems, including depressive mood and interest decline. The response options are “not at all,” “several days,” “more than half the days,” and “nearly every day,” scored as 0, 1, 2, and 3, respectively. The total score indicates different levels of depressive symptoms: minimal/no depression (0–4), mild (5–9), moderate (10–14), or severe (≥15) (11– 13).
3. The Generalized Anxiety Disorder scale (GAD-7): The GAD-7 scale is a recently developed 7-item tool based on DSM-IV criteria, which can easily screen anxiety symptoms (14). Participants are asked how often they were bothered by each symptom during the last 2 weeks, with a 4-point scale ranging from “not at all” (0 points) to “nearly every day” (3 points). The GAD-7 scale has been found to have good reliability among Chinese people (Cronbach's alpha = 0.90) (15, 16). The total score indicates different levels of anxious symptoms: minimal/no anxiety (0–4), mild (5–9), moderate (10–14), or severe (≥15).
4. The self-designed study-effect survey: This survey consists of ten questions, including (1). What do you think of the efficiency of the online-study during home quarantine compared with studying at school? Options: ①Higher; ②Almost the same; ③Lower. (2). How long do you study at home during quarantine every day? Options: ①More than 10 h; ②8–10 h; ③6–8 h; ④ <6 h. (3). Could you finish your homework on time? Options: ①Always; ②Often; ③Only sometimes; ④Never. (4). How is the interaction between you and your teachers during online-study compared with at school? Options: ①More interactive than before; ②Almost the same; ③Less interactive than before; ④Little interaction. (5) Are you disturbed by the external interference when studying at home during quarantine? Options: ①Never; ②Only sometimes; ③Often; ④Always. (6) Do you need parents' supervision on your study during quarantine? Options: ①Never; ②Only sometimes; ③Often; ④Always. (7) How much could you master from the online-study? Options: ①More than 90%; ②65–90%; ③40–65%; ④ <40%. (8). Are you tired of the online-study? Options: ①Never; ②Only sometimes; ③Often; ④Always. (9). Are you eager to study at school in a normal way? Options: ①Never; ②Only sometimes; ③Often; ④Always. (10). How is your relationship with your family during home quarantine? Options: ①Always harmonious; ②Not bad; ③Not quite good; ④Poor. For Question 1, each option represents 2 points, 1 point, 0 point, respectively. The options of remaining questions represent 3 points, 2 points, 1 point, 0 point, respectively, according to their own satisfaction of study-effect. We set the study-effect level based on the total score as follows: Excellent (>20), Good (16–20), Not good (11–15), or Poor (≤ 10).

### Investigation Approach

The Electronic “Questionnaire Star” tool (https://www.wjx.cn/) was used to send questionnaire and collect data from the participants. As a professional online survey platform, the “Questionnaire Star” has strengths in being efficient, costless, easy to learn and use, and has been applied in some investigations related to the Covid-19 Pandemic (10, 17, 18).

### Statistical Analysis

The statistical analyses were performed using IBM SPSS Statistics (version 21.0; IBM, Armonk, NY, USA). The categorical variables were expressed as the frequency (%), while the continuous variables were presented as mean ± SD. Differences in scores between male students and female students were assessed using the Independent samples *t*-test. Differences in scores among three grades were assessed using the One-way ANOVA. Spearman's correlation coefficient, *r*, was used to evaluate the association between depression level, anxiety level, as well as study-effect survey scores for exploratory analysis. A two-tailed *P* < 0.05 was considered statistically significant.

## Result

### Demographic Characteristics

A total of 1,020 senior high school students submitted their questionnaires, but two of them were excluded because the ages were fabricated. Finally, 1,018 qualified questionnaires were obtained, and the final recovery rate was 99.8%. The average age of the respondents was 16.61 ± 1.06 (years), 53.5% of them were female. The respondents all lived in Shandong Province; 81.4% lived in the city. Eight students got a history of close contact to SARS-CoV-2. We also classified the participants by grade. The detailed characteristics of the subjects were shown in Table 1.

**Table 1:** Demographic characteristics of the sample.

**Variables**	**All**	**Grade one**	**Grade two**	**Grade three**
Total number	1,018	496	267	255
**Gender**				
Male, *n* (%)	473 (46.5)	232 (46.8)	122 (45.7)	119 (47.7)
Female, *n* (%)	545 (53.5)	264 (53.2)	145 (54.3)	136 (53.3)
Age (years)	16.61 ± 1.06	15.80 ± 0.68	17.04 ± 0.59	17.76 ± 0.63
**Current residence**				
City, *n* (%)	829 (81.4)	406 (81.9)	209 (78.3)	214 (83.9)
Rural areas, *n* (%)	189 (18.6)	90 (18.1)	58 (21.7)	41 (16.1)
**History of close contact to SARS-CoV-2**				
Yes, *n* (%)	8 (0.8)	3 (0.6)	3 (1.1)	2 (0.8)
No, *n* (%)	1,010 (99.2)	493 (99.4)	264 (98.9)	253 (99.2)

### Depressive Symptoms

In total, the prevalence of depressive symptoms was 52.4% from mild to severe. The rate of all students with moderate-to-severe depressive symptoms was 17.6%. The rate of severe symptoms was 4.4%. From the perspective of gender, the depressed rate and the PHQ-9 mean score of female students were higher than male students (55.6 vs. 48.6%, and 5.82 ± 4.69 vs. 5.12 ± 4.92, respectively). In terms of the grade, grade one students exhibited the highest depression rate (60.1 vs. 45.3% and 44.7%). The PHQ-9 mean score was also higher in grade one students than the other two grades (6.11 ± 4.90 vs. 4.92 ± 4.54 and 4.89 ± 4.75). The detailed results were shown in Table 2.

**Table 2:** The rate of different severities of depressive symptoms in high school students assessed by PHQ-9.

**Variables**	**Gender**		***P***	**Grade**			***P***	**All**
	**Male**	**Female**		**Grade one**	**Grade two**	**Grade three**		**(*n* = 1,018)**
	**(*n* = 473)**	**(*n* = 545)**		**(*n* = 496)**	**(*n* = 267)**	**(*n* = 255)**		
Mean score	5.12 ± 4.92	5.82 ± 4.69	0.021^*^	6.11 ± 4.90	4.92 ± 4.54	4.89 ± 4.75	0.0003^**^	5.49 ± 4.81
Minimal/	243 (51.4)	242 (44.4)		198 (39.9)	146 (54.7)	141 (55.3)		485 (47.6)
No depression								
Mild	158 (33.4)	196 (36.0)		196 (39.5)	82 (30.7)	76 (29.8)		354 (34.8)
Moderate	51 (10.8)	83 (15.2)		73 (14.7)	31 (11.6)	30 (11.8)		134 (13.2)
Severe	21 (4.4)	24 (4.4)		29 (5.8)	8 (3.0)	8 (3.1)		45 (4.4)
Mild to severe	230 (48.6)	303 (55.6)		298 (60.1)	121 (45.3)	114 (44.7)		533 (52.4)

*PHQ-9, Patient Health Questionnaire 9-item*.

* *P < 0.05*.

** *P < 0.001*.

Among the ten depressive symptoms, the most common one is “Feeling tired or having little energy” (59.8%). The least common one is “Poor appetite or overeating” (31.1%). The detailed results were shown in Supplementary Table 1.

### Anxious Symptoms

The rate of all students with mild-to-severe anxiety symptoms was 31.4%. The prevalence of anxious symptoms was 4.6% from mild to severe. The rate of severe symptoms was 1.1%. From the perspective of gender, female students got a higher rate of anxiety than male (35.0 vs. 27.3%). In terms of the grade, the depressed rate of grade one students was slightly higher than the other two grades (33.1 vs. 31.1% and 28.6%). Grade one students also got the highest mean GAD-7 score (3.48 ± 3.48). The detailed results were shown in Table 3.

**Table 3:** The rate of different severities of anxious symptoms in high school students assessed by GAD-7.

**Variables**	**Gender**		***P***	**Grade**			***P***	**All**
	**Male**	**Female**		**Grade one**	**Grade two**	**Grade three**		**(*n* = 1,018)**
	**(*n* = 473)**	**(*n* = 545)**		**(*n* = 496)**	**(*n* = 267)**	**(*n* = 255)**		
Mean score	2.90 ± 3.18	3.56 ± 3.28	0.001^*^	3.48 ± 3.48	3.20 ± 2.88	2.87 ± 3.12	0.048^*^	3.25 ± 3.25
Minimal/	344 (72.7)	354 (65.0)		332 (66.9)	184 (68.9)	182 (71.4)		698 (68.6)
No depression								
Mild	112 (23.7)	161 (29.5)		134 (27.0)	75 (28.1)	64 (25.1)		273 (26.8)
Moderate	11 (2.3)	25 (4.6)		24 (4.8)	5 (1.9)	7 (2.7)		36 (3.5)
Severe	6 (1.3)	5 (0.9)		6 (1.2)	3 (1.1)	2 (0.8)		11 (1.1)
Mild to severe	129 (27.3)	191 (35.0)		164 (33.1)	83 (31.1)	73 (28.6)		320 (31.4)

*GAD-7, Generalized Anxiety Disorder scale*.

* *P < 0.05*.

Among the seven anxious symptoms, the most common one is “Being so restless that it is hard to sit still” (60.6%). The least common one is “Becoming easily annoyed or irritable” (26.9%). Nearly half (46.8%) of the students were not able to stop or control worrying. The detailed results were shown in Supplementary Table 2.

### Comorbid Depression and Anxiety Symptoms

The prevalence of comorbid depressive and anxiety symptoms among the students was 26.8%. Female students got a higher rate than male (30.8 vs. 22.2%). Grade one students got a higher rate than the other two grades (30.6 vs. 24.7% and 21.6%). See Table 4.

**Table 4:** The rate of comorbid depression and anxiety symptoms in high school students.

**Variables**	**Male**	**Female**	**Grade one**	**Grade two**	**Grade three**	**All**
	**(*n* = 473)**	**(*n* = 545)**	**(*n* = 496)**	**(*n* = 267)**	**(*n* = 255)**	**(*n* = 1,018)**
Comorbid depression and anxiety symptoms (Mild to severe)	105 (22.2)	168 (30.8)	152 (30.6)	66 (24.7)	55 (21.6)	273 (26.8)

### Online-Study Effect Evaluation

Nearly half (47.4%) of the students were not satisfied with their online-study effect (“poor” or “not good” for the total score). Male students and female students were nearly the same, while grade three students felt better with their study effect than the other grades. More than half (56.9%) of them considered that the efficiency of the online-study during home quarantine was lower than studying at school (Question 1). Nearly half (45.6%) of the students were always eager to study at school in a normal way (Question 9). Most of the students (85.0%) had a good relationship with their family during quarantine (Question 10). See Table 5, and Supplementary Table 3 for more details.

**Table 5:** The self-evaluation of online-study effect in high school students.

**Variables**	**Gender**		***P***	**Grade**			***P***	**All**
	**Male**	**Female**		**Grade one**	**Grade two**	**Grade three**		**(*n* = 1,018)**
	**(*n* = 473)**	**(*n* = 545)**		**(*n* = 496)**	**(*n* = 267)**	**(*n* = 255)**		
Mean score	15.27 ± 4.67	15.68 ± 4.49	0.158	15.42 ± 4.51	14.92 ± 4.67	16.23 ± 4.54	0.004^*^	15.49 ± 4.58
Excellent	56 (11.8)	78 (14.3)		59 (11.9)	33 (12.4)	42 (16.5)		134 (13.2)
Good	185 (39.1)	216 (39.6)		201 (40.5)	89 (33.3)	111 (43.5)		401 (39.4)
Not good	160 (33.8)	178 (32.7)		167 (33.7)	97 (36.3)	74 (29.0)		338 (33.2)
Poor	72 (15.2)	73 (13.4)		69 (13.9)	48 (18.0)	28 (11.0)		145 (14.2)

* *P < 0.05*.

### Correlations Between Depressive Symptoms, Anxious Symptoms and Online-Study Effect

**Figure 1:**
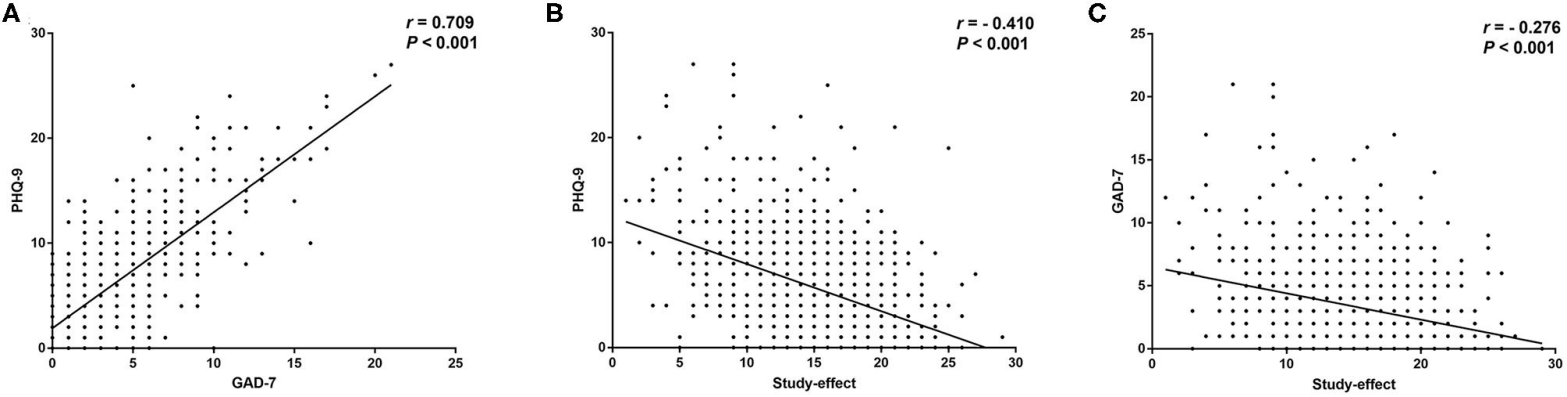
Correlations between depressive symptoms, anxious symptoms and online-study effect. **(A)** Correlation between PHQ-9 and GAD-7. **(B)** Correlation between PHQ-9 and online-study effect. **(C)** Correlation between GAD-7 and online-study effect.

The PHQ-9 score had a strong positive correlation with the GAD-7 score in all students (*r* = 0.709, *P* < 0.001) (Figure 1A). The PHQ-9 score had a moderate negative correlation with the study-effect survey score (*r* = −0.410, *P* < 0.001) (Figure 1B), and the GAD-7 score had a weak negative correlation with the study-effect survey score (*r* = −0.276, *P* < 0.001) (Figure 1C).

## Discussion

This epidemiological survey indicated that during the COVID-19 pandemic, the prevalence of depressive and anxious symptoms of high school students in Shandong Province was 52.4 and 31.4% from mild to severe, respectively. The prevalence of comorbid depressive and anxiety symptoms was 26.8%. The PHQ-9 score was strongly positively correlated with the GAD-7 score in all students. Girls and grade one students seem to be more likely to suffer from psychological problems. Nearly half of the students were not satisfied with their online-study effect facing with school closures, and were always eager to studying at school in a normal way. Our findings provided supplementary perspective to comprehensively understand the psychological status of Chinese populations during the COVID-19.

The prevalence of depression in this present study is higher than pre-COVID-19 times (7, 19). For students in a state of depression or anxiety, most of them were mild or moderate, and a few of them were severe. All the participants in this study were in Shandong Province, a place located on the east coast of China. As a relatively developed province, Shandong got the second largest population and the third largest gross domestic product (GDP) in China (http://tjj.shandong.gov.cn/). The urban population accounts for 60.58% of the total population (http://www.shandong.gov.cn). The population density is 634 people/square kilometer. The three high schools in this present study lie in the city so most of the students were urban residents. Though the epidemic has been well controlled in Shandong Province with few confirmed cases and low mortality (787 confirmed cases and 7 deaths as of April 30th) in over one hundred million populations (http://wsjkw.shandong.gov.cn/), it still brings panic and pressure to general people, and fears and stresses might be contagious among family members. According to our GAD-7 results, 46.8% of the students felt “not able to stop or control worrying” and 33.4% felt “worried too much about different things.” During the prolonged time of isolation, some families lost their source of income because of the epidemic. Economic downturns are associated with an increase in mental health problems for children and adolescents, which might be affected by the ways that economic downturns affect adult unemployment, adult mental health and child maltreatment (5). Students themselves might feel depressed and anxious about struggling to pay their tuition fees or maintain stability in their life (20). Furthermore, during quarantine, depressive and anxious symptoms are more likely to occur and worsen in the absence of interpersonal communication (21, 22). During adolescence, young people grow in independence and begin to prioritize connections with peers over parents (23). Normal and healthy social activities are significant to stable emotions and good psychological status.

In our study, 47.4% of all students were not satisfied with their online-study effect at home. Correlation analysis indicated that the study-effect was closely related with their depressive and anxious symptoms. High school students were facing too much academic pressure from the college entrance examination (10, 24). According to our PHQ-9 and GAD-7 results, the most common depression symptom and anxiety symptom were “Feeling tired or having little energy” and “Being so restless that it is hard to sit still.” This situation might be worsened due to school closures with unsatisfactory remote learning. As shown in our findings, 56.9% of the students considered that the efficiency of the online-study during home quarantine was lower than studying at school, 57.0% of them studied for < 8 h at home every day (less than school days). Most of the students (71.0%) thought that the interactions between student and teacher were less than before or little interaction existed. Nearly half of the students were always eager to study at school in a normal way. The contradiction between pandemic school closures and the demands of studying normally might lead to aggravating mental health problems.

From the perspective of gender, both the depression and anxiety rate and the symptom severity of female students were higher than male students. This is consistent with former studies which found that female students have suffered from greater psychological impact, as well as higher levels of stress, anxiety, and depressive symptoms during the COVID-19 pandemic (10, 25). Previous studies indicated that stress exposure would increase rates of mental problems in adults, particularly in females (26), and females are also more susceptible to insomnia (27). Thus, female gender might be a higher risk factor for depression and anxiety symptoms specific for this study. When it comes to the grade, grade one students got a higher rate of depression and anxiety and severer symptoms than the other two grades. This is inconsistent with a previous study which demonstrated that the higher the grade, the greater the risk of depressive and anxious symptoms (10). This might be due to the heterogeneity of different samples. They conducted their study in March during the early outbreak of COVID-19. At the time we started to collect the data (May 1st), the epidemic had tended to be moderated and had been spread at a much slower pace. High schools in Shandong Province had been partially reopened with grade three students already went back to school normally for 2 weeks, while grade one and grade two students were still in quarantine and studying online. This might lead to a biased result. Besides, with the age growing older, the students could be better at managing pressures and regulating emotions. Some researchers found younger age might be potential risk factors for the psychological problems of the public during COVID-19 (16). This is also in line with the findings that psychiatric morbidities were associated with younger age and increased self-blame during the SARS outbreak (28).

Our findings could provide significant guidance for the development of psychological support strategies in high school students, especially during the period of school reopening. High-risk groups such as female students and grade one student deserve special concerns. Attentions should also be paid to the potential effects on individuals such as posttraumatic stress disorder. The Ministry of Education of China has promoted several suggestions for protecting mental health in primary school, middle school and high school (http://www.moe.gov.cn/jyb_xwfb), mainly including improving the students' learning ability and adaptability in the new semester; evaluating mental status of teachers and students; identifying the immediate psychological needs for student individually; providing psychological interventions for students with psychological distress; relieving the teachers' pressures and guiding them to carry out teaching in an orderly way; strengthening communications between school and family, and assisting them to establish a harmonious relationship. In a word, the society, school and family should take up their responsibilities to maintain a healthy psychological status of students during the COVID-19 epidemic.

The COVID-19 epidemic brings physical risks and psychological challenges to high school students. Meanwhile, the pandemic offers an opportunity for young people to develop and hone their resilience and adaptability, and appreciate the value of social responsibility and self-sacrifice for the protection of the most vulnerable (23). We should recognize the efforts and contributions of them in this global crisis, and give sufficient attentions to their physical and mental health.

There are some limitations in this study to be addressed. Firstly, the samples were restricted in one province. Shandong is a relatively developed coastal province and most students were city residents. Our findings may not reflect the circumstances in broader regions. Secondly, a self-designed questionnaire for study effect was used, which might have a certain result deviation. Thirdly, due to the limitation of online questionnaire, the results were not always consistent with professional evaluation. Fourthly, it would be more meaningful to explore the students' family characteristics and possible correlation to their psychological status and requirement for high level education, as well as the association between the online study effect and teachers' mental health. Future studies may collect information including parental educational level, socioeconomic status, parental work and the teachers' psychological status to provide a comprehensive perspective. Fifthly, as a convenience sampling study through the internet, we didn't calculate the sample size for a more standard statistic. Last but not least, this was a cross-sectional study. It would be better to follow up the change of the students' psychological status to provide necessary support.

## Conclusion

Our findings indicated that more than half of the high school students suffered from depressive symptoms, and nearly one-third of them suffered from anxious symptoms. And from moderate to severe severity level, the rates of depressive symptoms and anxious symptoms were 17.6 and 4.6%. Quite a number of them were not satisfied with their online-study effect during quarantine, and the study effect was correlated with their psychological status. Sufficient attentions should be paid to the mental health of the high school students.

## Data Availability Statement

The raw data supporting the conclusions of this article will be made available by the authors, without undue reservation.

## Ethics Statement

The studies involving human participants were reviewed and approved by the Ethics Committee of the Jining Psychiatric Hospital. Written informed consent from the participants' legal guardian/next of kin was not required to participate in this study in accordance with the national legislation and the institutional requirements.

## Author Contributions

CZ designed the study and revised the manuscript. ZZ wrote the initial manuscript. ZZ, AZ, MY, and CY collected the data and undertook the statistical analysis. JZ and HZ assisted with data collection and statistical analysis and interpreted the data. SD modified the paper. All authors contributed to the article and approved the submitted version.

## Conflict of Interest

The authors declare that the research was conducted in the absence of any commercial or financial relationships that could be construed as a potential conflict of interest.
