# Supplementary material for: Prevalence of Depression and Anxiety Symptoms of High School Students in Shandong Province During the COVID-19 Epidemic
Source: Front Psychiatry. 2020 Dec 21;11:570096. doi: 10.3389/fpsyt.2020.570096 (PMC7779476; doi:10.3389/fpsyt.2020.570096)
Supplement: Supplementary file 1 [file Data_Sheet_1.doc]

**Supplementary Material**

**Table S1** The rate of different depressive symptoms in high school students assessed by PHQ-9. [*N* (%)]

| Items | Not at all | Several days | More than half  the days | Nearly everyday |
| --- | --- | --- | --- | --- |
| Little interest or pleasure in doing things | 545 (53.5) | 411 (40.4) | 44 (4.3) | 18 (1.8) |
| Feeling down, depressed, or hopeless | 452 (44.4) | 492 (48.3) | 60 (5.9) | 14 (1.4) |
| Trouble falling or staying asleep, or sleeping too much | 594 (58.3) | 350 (34.4) | 56 (5.5) | 18 (1.8) |
| Feeling tired or having little energy | 409 (40.2) | 457 (44.9) | 121 (11.9) | 31 (3.0) |
| Poor appetite or overeating | 701 (68.9) | 278 (27.3) | 24 (2.4) | 15 (1.5) |
| Feeling bad about yourself - or that you are a failure or have let yourself or your family down | 501 (49.2) | 417 (41.0) | 70 (6.9) | 30 (2.9) |
| Trouble concentrating on things, such as reading the newspaper or watching television | 430 (42.2) | 454 (44.6) | 104 (10.2) | 30 (2.9) |
| Moving or speaking so slowly that other people could have noticed. Or the opposite - being so fidgety or restless that you have been moving around a lot more than usual | 424 (41.7) | 470 (46.2) | 96 (9.4) | 28 (2.8) |
| Thoughts that you would be better off dead, or of hurting yourself in some way | 545 (53.5) | 396 (38.9) | 63 (6.2) | 14 (1.4) |

PHQ-9: Patient Health Questionnaire 9-item.

**Table S2** The rate of different anxious symptoms in high school students assessed by GAD-7. [*N* (%)]

| Items | Not at all | Several days | More than half  the days | Nearly everyday |
| --- | --- | --- | --- | --- |
| Feeling nervous, anxious or on edge | 607 (59.6) | 360 (35.4) | 34 (3.3) | 17 (1.7) |
| Not being able to stop or control worrying | 542 (53.2) | 429 (42.1) | 34 (3.3) | 13 (1.3) |
| Worrying too much about different things | 687 (66.6) | 251 (24.7) | 51 (5.0) | 29 (2.8) |
| Trouble relaxing | 688 (67.6) | 249 (24.5) | 53 (5.2) | 28 (2.8) |
| Being so restless that it is hard to sit still | 401 (39.4) | 480 (47.2) | 108 (10.6) | 29 (2.8) |
| Becoming easily annoyed or irritable | 744 (73.1) | 243 (23.9) | 25 (2.5) | 6 (0.6) |
| Feeling afraid as if something awful might happen | 729 (71.6) | 255 (25.0) | 29 (2.8) | 5 (0.5) |

GAD-7: Generalized Anxiety Disorder scale.

**Table S3 The self-evaluation of online-study effect in high school students. [*N* (%)]**

| Items | ① | ② | ③ | ④ |
| --- | --- | --- | --- | --- |
| What do you think of the efficiency of the online-study during home quarantine compared with studying at school? | 58 (5.7) | 381 (37.4) | 579 (56.9) | - |
| How long do you study every day during home quarantine? | 96 (9.4) | 341 (33.5) | 373 (36.6) | 208 (20.4) |
| Could you finish your homework on time? | 342 (33.6) | 492 (48.3) | 154 (15.1) | 30 (2.9) |
| How is the interaction between you and teacher during online-study compared with at school? | 36 (3.5) | 260 (25.5) | 416 (40.9) | 306 (30.1) |
| Are you disturbed by the external interference when studying at home during quarantine? | 178 (17.5) | 568 (55.8) | 209 (20.5) | 63 (6.2) |
| Do you need parents' supervision on study during quarantine? | 358 (35.2) | 445 (43.7) | 136 (13.4) | 79 (7.8) |
| How much could you master from the online-study? | 134 (13.2) | 612 (60.1) | 218 (21.4) | 54 (5.3) |
| Are you tired of the online-study? | 191 (18.8) | 480 (47.2) | 227 (22.3) | 120 (11.8) |
| Are you eager to study at school in a normal way? | 42 (4.1) | 192 (18.9) | 320 (31.4) | 464 (45.6) |
| How is your relationship with family during home quarantine? | 452 (44.4) | 413 (40.6) | 126 (12.3) | 27 (2.7) |
